# A New Approach to Understanding Cancer-Related Fatigue: Leveraging the 3P Model to Facilitate Risk Prediction and Clinical Care

**DOI:** 10.3390/cancers14081982

**Published:** 2022-04-14

**Authors:** Alix G. Sleight, Sylvia L. Crowder, Jacek Skarbinski, Paul Coen, Nathan H. Parker, Aasha I. Hoogland, Brian D. Gonzalez, Mary C. Playdon, Steven Cole, Jennifer Ose, Yuichi Murayama, Erin M. Siegel, Jane C. Figueiredo, Heather S. L. Jim

**Affiliations:** 1 Department of Physical Medicine & Rehabilitation, Cedars-Sinai Medical Center, Los Angeles, CA 90048, USA; alixsleight.warner@cshs.org; 2 Center for Integrated Research in Cancer and Lifestyle, Cedars-Sinai Medical Center, Los Angeles, CA 90048, USA; 3 Department of Health Outcomes and Behavior, Moffitt Cancer Center, Tampa, FL 33601, USA; sylvia.crowder@moffitt.org (S.L.C.); nathan.parker@moffitt.org (N.H.P.); aasha.hoogland@moffitt.org (A.I.H.); brian.gonzalez@moffitt.org (B.D.G.); 4 Division of Research, Kaiser Permanente Northern California, Oakland, CA 94501, USA; jacek.skarbinski@kp.org; 5 Department of Infectious Diseases, Oakland Medical Center, Kaiser Permanente Northern California, Oakland, CA 94501, USA; 6 Physician Researcher Program, Kaiser Permanente Northern California, Oakland, CA 94501, USA; 7 The Permanente Medical Group, Kaiser Permanente Northern California, Oakland, CA 94501, USA; 8 AdventHealth Orlando, Translational Research Institute, Orlando, FL 32804, USA; paul.coen@adventhealth.com; 9 Department of Nutrition and Integrative Physiology, University of Utah, Salt Lake City, UT 84044, USA; mary.playdon@hci.utah.edu; 10 Department of Cancer Control and Population Sciences, Huntsman Cancer Institute, University of Utah, Salt Lake City, UT 84044, USA; 11 Department of Psychiatry & Biobehavioral Sciences and Medicine, University of California, Los Angeles, CA 90001, USA; coles@ucla.edu; 12 Department of Population Sciences, University of Utah, Salt Lake City, UT 84044, USA; jennie.ose@hci.utah.edu; 13 Huntsman Cancer Institute, University of Utah, Salt Lake City, UT 84044, USA; 14 Department of Medicine, Samuel Oschin Comprehensive Cancer Institute, Cedars-Sinai Medical Center, Los Angeles, CA 90048, USA; yuichi.murayama@cshs.org (Y.M.); jane.figueiredo@cshs.org (J.C.F.); 15 Department of Cancer Epidemiology, Moffitt Cancer Center, Tampa, FL 33601, USA; erin.siegel@moffitt.org

**Keywords:** fatigue, metabolomics, survivorship

## Abstract

**Simple Summary:**

For the growing number of cancer survivors worldwide, fatigue presents a major hurdle to function and quality of life. Treatment options for cancer-related fatigue are still emerging, and our current understanding of its etiology is limited. In this paper, we describe a new application of a comprehensive model for cancer-related fatigue: the predisposing, precipitating, and perpetuating (3P) factors model. We propose that the 3P model may be leveraged—particularly using metabolomics, the microbiome, and inflammation in conjunction with behavioral science—to better understand the pathophysiology of cancer-related fatigue.

**Abstract:**

A major gap impeding development of new treatments for cancer-related fatigue is an inadequate understanding of the complex biological, clinical, demographic, and lifestyle mechanisms underlying fatigue. In this paper, we describe a new application of a comprehensive model for cancer-related fatigue: the predisposing, precipitating, and perpetuating (3P) factors model. This model framework outlined herein, which incorporates the emerging field of metabolomics, may help to frame a more in-depth analysis of the etiology of cancer-related fatigue as well as a broader and more personalized set of approaches to the clinical treatment of fatigue in oncology care. Included within this review paper is an in-depth description of the proposed biological mechanisms of cancer-related fatigue, as well as a presentation of the 3P model’s application to this phenomenon. We conclude that a clinical focus on organization risk stratification and treatment around the 3P model may be warranted, and future research may benefit from expanding the 3P model to understand fatigue not only in oncology, but also across a variety of chronic conditions.

## 1. Introduction

Cancer-related fatigue is defined by the National Comprehensive Cancer Network (NCCN) as a distressing, persistent sense of physical, emotional, and/or cognitive exhaustion related to cancer that is not proportional to activity and interferes with functioning [1]. Moderate to severe fatigue affects up to 90% of patients during chemotherapy and approximately 30–40% years after treatment completion [1,2]. Unlike typical fatigue, cancer-related fatigue tends to be more severe, distressing, and unlikely to be relieved by rest [3]. Patients describe it as “devastating”, “never-ending” and “totally consuming” [4,5]. Fatigue is associated with worse quality of life and lower likelihood of returning to normal daily activities, including work [2,6,7,8,9]. 

Treatment options for cancer patients with fatigue are limited. Behavioral and psychosocial interventions demonstrate benefit [10,11,12,13,14] but tend to be time intensive, limiting uptake, compliance, and maintenance. Medications to treat sleep problems such as paroxetine, sertraline, modafinil, and armodafinil have shown limited benefit for cancer-related fatigue in randomized trials [15,16,17,18,19,20]. Evidence is mixed for methylphenidate, which may be poorly tolerated [21,22,23,24]. Because of the limited benefit of pharmacotherapy and since patients often prefer to avoid additional medications, behavioral treatment options for cancer-related fatigue are urgently needed [25,26]. A major gap impeding development of new treatments is an inadequate understanding of the complex biological, clinical, demographic, and lifestyle mechanisms underlying fatigue [6]. In this paper, we describe a new application of a comprehensive model for a better understanding of cancer-related fatigue: the predisposing, precipitating, and perpetuating (3P) factors model. This model framework outlined herein, which incorporates the emerging field of metabolomics, may help to frame a more in-depth analysis of the etiology of cancer-related fatigue as well as a broader and more personalized set of approaches to the clinical treatment of fatigue in oncology care.

## 2. Proposed Biological Mechanisms of Cancer-Related Fatigue 

The pathophysiology of cancer-related fatigue is thought to be multifactorial [27,28]. Despite this complexity, the large majority of studies on the biological mechanisms of cancer-related fatigue have focused on immune and inflammatory variables, which are hypothesized to induce fatigue via the effect of inflammatory mediators on brain systems involved in “sickness behaviors.” Specific variants include genes regulating inflammation (e.g., IL6, TNFA, and IL1) [29], inflammatory gene expression profiles (e.g., increased NF-kB) [30,31,32], and circulating markers of inflammation (e.g., IL-1, TNFA, CRP, and IL-6) [32,33,34,35,36,37,38,39,40,41,42,43,44,45,46,47,48,49,50,51,52,53]. Inflammation is linked with the dysregulation of biochemical and physiological systems including peripheral (muscles and tissues) and central mechanisms (central nervous system) [54] and may cause fatigue through cytokine dysregulation, hypothalamic–pituitary–adrenal (HPA) axis dysfunction, 5-hydroxy-tryptophan (5-HT) neurotransmitter dysregulation, circadian rhythm disruption, alterations in adenosine triphosphate (ATP), muscle metabolism, and vagal afferent activation [28,54,55]. Recent research has suggested that agents with anti-inflammatory properties (i.e., non-steroidal anti-inflammatory drugs or NSAIDs, bupropion) could offer a safe, inexpensive, and widely-available means to revolutionize the treatment of cancer-related fatigue [56,57,58,59]. These findings suggest that pathophysiologic pathways and genetic mechanisms hold promise for the identification of new causal mechanisms and potential treatment targets; however, these mechanisms are poorly understood. In addition, current models of pathophysiology do not take into account the complex psychosocial and behavioral factors that may also play a major role in cancer-related fatigue.

## 3. An Alternative Model of Cancer-Related Fatigue: The 3P Factors Model

**Figure 1:**
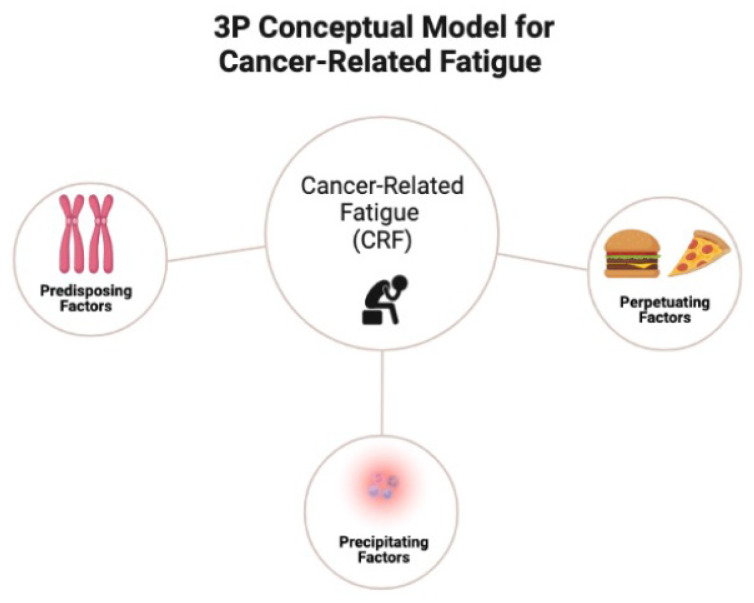
The 3P conceptual model for cancer-related fatigue.

The most notable framework previously proposed to describe complex disease processes is the biopsychosocial model, an inter-disciplinary model that looks at the interconnection between biology, psychology, and socioenvironmental factors [60]. While the biopsychosocial model has played a crucial role in counteracting biological reductionism and progressing towards a more holistic philosophy of human health, it lacks the granularity necessary to understand how various factors contribute to disease [61]. In contrast, as shown in Table 1, the 3P model can be utilized to describe the complex biological and psychological processes underlying cancer-related fatigue. The 3P model postulates that predisposing factors place patients at risk of developing baseline fatigue (e.g., 1. biobehavioral: age, biological sex, genetic variants, metabolomics, inflammation, body composition, nutritional quality, circadian disruption, and co-morbidities; 2. psychosocial: depressed mood, anxiety, insomnia, and perceived stress); precipitating factors spur the onset of fatigue (e.g., changes in metabolism and inflammation due to cancer and/or chemotherapy and treatment-related factors: systemic therapy and radiotherapy); and perpetuating factors worsen fatigue or cause it to become chronic (e.g., poor sleep, physical inactivity, and poor diet). The 3P model (Figure 1) has been suggested for better understanding fatigue [62] and successfully applied to other chronic conditions including sleep and pain [24,25]. 

### 3.1. Predisposing Factors 

Patient characteristics conceptualized as predisposing factors in cancer-related fatigue include biological sex [63], genetics [34], body composition (e.g., body fat and low muscle mass) [64,65,66], and viral exposures [67,68]. Additionally, circadian rhythms could play a significant role in the etiology of fatigue through the modulation of arousal and sleep [54].

Predisposing risk factors for cancer-related fatigue include poor performance status, chemoradiotherapy, female sex, insomnia, neuroticism, pain, and depression [69]. In cancer-related fatigue, the role of genetic variation remains unclear. Twin studies have shown the heritability of fatigue to be between 6% and 50%, with a higher concordance in monozygotic twins than dizygotic twins. Some preliminary studies have identified sets of inflammation-related genetic polymorphisms that are associated with increased fatigue in cancer patients [34], but the generality of these effects remains to be determined. Genome-wide association studies (GWAS) in fatigue-related diseases have identified variants in genes involved in cognition and circadian rhythms [70,71,72]. We propose that genetic variants altering metabolic including inflammatory traits may also be associated with cancer-related fatigue through inflammation pathways. Publicly available lists of high-scoring genetic–metabolomic associations known as “genetically influenced metabotypes” include several variants located in or near genes encoding enzymes central to human lipid metabolism, including polyunsaturated fatty acid biosynthesis (e.g., FADS1, ELOVL2) and biosynthesis of phospholipids (e.g., SPT16A) [73], which have not been explored in cancer-related fatigue. Similarly, “genetically influenced inflammotypes” [74] can be identified by inflammatory-based genome-wide association studies (iWAS), but also have not yet been explored among cancer patients with fatigue. 

Previous viral exposure—for example, to Epstein–Barr virus, human herpesvirus, Lyme disease, or COVID-19—may predispose individuals to fatigue through cell alterations, hyperinflammation, mitochondrial modulation, and autoimmunity, although research in this area is lacking [75,76]. Additionally, anthropometry measurements (e.g., obesity) have been associated with links in apnea, sleep quality, and inflammatory biomarkers.

### 3.2. Precipitating Factors 

Factors that may initially precipitate the development of cancer-related fatigue remain unclear, though likely include metabolic dysregulation (alterations in metabolic genes and regulatory pathways), as well as inflammation (overproduction of pro-inflammatory cytokines) and accelerated cellular aging (e.g., the premature shortening of telomeres and altered DNA methylation) due to cancer treatment. For example, chemotherapy is known to accelerate aging [77,78]. Chemotherapy may also damage mitochondria in muscle and deconditioning of muscle that may contribute to perceptions of fatigue [79,80,81,82].

Multiplicative interactions between precipitating factors may also exist. Studies investigating muscle fatigue in cancer patients show metabolic dysregulation, including energy, lipid, and amino acid metabolism [83,84,85,86]. Furthermore, evidence supports that chemotherapy may damage mitochondria in muscle that in turn increases fatigue, and the deconditioning of muscle further contributes to perceptions of fatigue. In particular, studies have focused on tryptophan catabolism [87,88,89]. An essential amino acid, tryptophan drives de novo synthesis of serotonin and niacin. Serotonin modulates behavioral and neuropsychological processes and niacin produces NAD, a co-factor crucial for energy homeostasis that is linked with aging and circadian regulation (SIRT1). Trials modifying tryptophan have demonstrated reductions in physical and mental fatigue following endurance exercise [90]. Furthermore, metabolic disturbances related to chronic fatigue syndrome have included alterations in 20 metabolic pathways including sphingolipids, phospholipids, purine, cholesterol, microbial metabolites, pyroline-5-carboxylate, riboflavin, amino acids, peroxisomal and mitochondrial metabolism [91]. All are directly regulated by redox or the availability of NADPH, highlighting the importance of the mitochondria, cellular organelles that produce energy [91]. Sphingolipids and phospholipids accounted for almost 70% of the variation in metabolic phenotype in a study of 84 patients with chronic fatigue syndrome, and differences among males and females were observed. Area under the receiver operator characteristic curve analysis showed accuracies in predicting fatigue of 94% (95% CI = 84–100%) for males and 96% (95% CI = 86–100%) for females. Three other metabolomics studies of fatigue-associated diseases support the key role of sphingolipids and phospholipids in addition to irregularities in energy, amino acid, and nucleotide metabolism [92,93,94]. The alterations in sphingolipids may be related to impaired lipid metabolism and mitochondria energetics, with evidence suggesting that PPAR suppression in the muscle of cancer patients could mediate this [81,95,96]. Furthermore, in vitro studies have demonstrated that ceramides induce oxidant production in the mitochondria, have specific effects in certain tissues (e.g., adipocyte ceramides and inflammation) and increase oxidant activity [97], depressing muscle fiber force and exacerbating muscle fatigue [98]. While a number of pathological pathways have been identified as playing a role in cancer-related fatigue, it is possible that different mechanisms are responsible for different dimensions of fatigue (e.g., mental fatigue vs. physical fatigue). Further delineation of unique dimensions of fatigue associated with each pathway will assist in the identification of new intervention targets for the specific type of fatigue experienced. 

### 3.3. Perpetuating Factors 

Perpetuating factors are conceptualized as characteristics and behaviors that may worsen or prolong fatigue including poor dietary pattern, irregular meal timing [99,100], physical inactivity [101], and poor sleep [102,103,104,105,106,107]. Previous research suggests that anti-inflammatory dietary patterns, such as prudent and Mediterranean diets, offer a plausible mechanism to mitigate cancer-related fatigue through reducing inflammation and improving body composition [108,109,110]. The key components of the Mediterranean dietary pattern include high intake of vegetables, fruits, whole grains, legumes, and nuts; moderate intake of seafood and red wine; and olive oil as the main fat source [111,112]. Anti-inflammatory dietary patterns are associated with improvements in the gastrointestinal (GI) microbiota and lessening of metabolic endotoxemia, defined as a 2- to 3-fold increase in circulating levels of bacterial endotoxin [113]. In comparison, pro-inflammatory dietary patterns, such as the Western dietary pattern, widely consumed in the United States, is characterized by high consumption of red and processed meats; high consumption of sugar-sweetened beverages and refined grains; and low consumption of fresh fruits, vegetables, and legumes [114,115,116]. Western diets contribute to metabolic endotoxemia through changes in the GI microbiome and bacterial fermentation end products, intestinal physiology and barrier function, and enterohepatic circulation of bile acids [113]. Additionally, the Western dietary pattern has been correlated with pro-inflammatory markers associated with cancer-related fatigue, including tumor necrosis factor (TNF)-α, C-reactive protein, interleukin (IL)-6, and IL-8 [117]. Dietary patterns promoting hyperinsulinemia and chronic inflammation, including the empirical dietary index for hyperinsulinemia (EDIH) and empirical dietary inflammatory pattern (EDIP), strongly influence risk of weight gain, type 2 diabetes, cardiovascular disease, and cancer [118]. The EDIH and EDIP have predicted concentrations of known insulinemic and inflammatory biomarkers, and the EDIH further predicted risk of future cancer [119].

In addition to evaluating dietary patterns based on self-reported questionnaires, the role of diet in cancer-related fatigue can be investigated through nutritional metabolomics, the study of food-related metabolites in a biofluid that can provide an objective measure of recent or habitual dietary intake [120]. Moreover, untargeted metabolomics offers a discovery tool to identify small molecules both influenced by dietary behavior and associated with disease, thus characterizing endogenous response to diet, and metabolic targets for dietary intervention for disease prevention. To our knowledge, nutritional metabolomics studies of cancer-related fatigue are yet to be implemented. The microbiota has been recognized to play a role in human disease [121], and the mechanisms by which these microorganisms contribute to host health have been extensively investigated over the past decade. The microbiome, specifically bacterial metabolites, has been linked with inflammation and oxidation. Two studies in mice have suggested that the gut microbiota produces metabolites from dietary tryptophan that regulate inflammation in the gut and central nervous system [122].

In terms of general lifestyle, prior research in fatigue-associated diseases highlights the role of lipid mediators including sphingolipids, phospholipids, and oxygenated polyunsaturated fatty acids (PUFAs) (oxylipins). Sphingolipid metabolites play key roles in the regulation of both trafficking and function of immune cells, and there are indications that sphingolipid metabolism might be altered by inflammation [123]. Ceramides, key sphingolipids, promote numerous inflammatory processes, including induction of macrophages and B cells [124]. Prior studies indicate alteration of ceramide metabolism among patients with chronic fatigue syndrome [92]. Intervention trials show that diet can lower ceramide levels [125]. In the PREDIMED study, a Mediterranean dietary intervention mitigated potential deleterious effects of elevated plasma ceramide concentrations on cardiovascular disease [126]. Similarly, omega-3 polyunsaturated fatty acid (n3-PUFA) is a common phospholipid, which plays an important role in immunomodulatory activities. Ceramides and its metabolites have been proposed as an intermediate link between over-nutrition and certain underlying abnormalities driving disease risk, insulin resistance and low-grade inflammation [127,128,129]. Data suggest beneficial effects of n3-PUFA in reducing fatigue in cancer patients [33,130,131,132,133]. N-3 PUFA therapy upregulates the muscle transcriptome, including several pathways that control mitochondrial function in both human [134], and animal studies [135,136,137], emphasizing the role of energy metabolism. Other metabolic pathways related to diet that might also contribute to fatigue include dysregulated tryptophan catabolism. Tryptophan is an amino acid metabolized into several molecules involved in energy production [87,88,89]. Potential interventions might target modulation of tryptophan-related molecules via administration of branched chain amino acids. In addition to endogenous metabolites, untargeted approaches may identify unexpected or novel exposures that might play an important role in cancer-related fatigue by implicating exogenously derived chemicals. Adherence to specific dietary patterns (e.g., time-restricted eating) may offer a novel, cost-effective strategy to reduce cancer-related fatigue while quantification of targeted metabolites may allow for a robust evaluation of metabolite changes in people with cancer-related fatigue over time [138].

There is an interaction of diet, physical activity, and sleep on many levels (e.g., behavioral, circadian, obesity, metabolic). Particularly, intermittent fasting regimens have been hypothesized to influence metabolic regulation via effects on (a) circadian biology, (b) the gut microbiome, and (c) modifiable lifestyle behaviors, such as sleep [100]. Evidence suggests that irregular meal timing may impact metabolic health. Specifically, eating more frequently, reducing evening energy intake, and fasting for longer nightly intervals may lower systemic inflammation and subsequently reduce breast cancer risk [99]. In another study examining associations between fasting duration, timing of first and last meals, and cardiometabolic endpoints using data from the National Health and Nutrition Examination Survey (NHANES), evidence suggested that there were beneficial effects on cardiometabolic health of starting energy consumption earlier in the day [139].

In addition to diet, physical inactivity and sleep disturbance represent key modifiable perpetuating factors associated with cancer-related fatigue [101]. In a systematic review and meta-analysis of randomized controlled trials, physical activity has been identified as effective for mitigating cancer-related fatigue in colorectal cancer [140]. Moderate-intensity aerobic exercise training and a combination of moderate-intensity aerobic and resistance training have reduced fatigue in patients with breast and prostate cancer, both during and following cancer therapy [141,142]. Reductions in fatigue from exercise training appear to result from both independent and supervised interventions [143], highlighting its potential applicability to a wide range of cancer patients and survivors. Similarly, sleep disturbance confers risk of cancer-related fatigue across various cancer diagnoses. A recent meta-analysis studying risk factors for cancer-related fatigue in 84 studies with 144,813 participants found that patients with insomnia had significantly higher odds of cancer-related fatigue [69]. Notably, the odds ratio for insomnia was higher than the odds ratio for treatment of chemoradiotherapy, although the magnitude of these effects was not formally compared. In patients with chronic myeloid leukemia receiving cognitive behavioral therapy for targeted-therapy-related fatigue, improvements in sleep and physical activity were associated with declines in fatigue [101]. Sleep disturbance and physical inactivity both contribute to known pathways for cancer-related fatigue (e.g., inflammation, circadian disruption) [144,145]. Emerging evidence suggests that sleep disturbance and physical inactivity may also implicate additional pathways, such as accelerated aging and gene expression through DNA methylation. For example, one study of 2078 women found that those with insomnia showed advanced biological age relative to chronological age [146]. In another study, individuals with insufficient sleep showed hypomethylation of DNA in regions associated with neuroplasticity and neurodegeneration [147]. Further research is necessary to elucidate the nuanced role of sleep and physical activity in cancer-related fatigue. Please see Table 1. 

## 4. Clinical Implications

Use of the 3P model can benefit clinical oncology practice by offering pathways to prevention and mitigation strategies for cancer-related fatigue. First, screening for predisposing factors of cancer-related fatigue may facilitate risk stratification of cancer patients at the time of diagnosis. Risk stratification has been endorsed by experts in oncology healthcare delivery science as a personalized approach to care in which survivors are triaged to distinct care pathways based on their individual needs [148]. For example, patients who display a genetic predisposition to cancer-related fatigue and fit a predetermined risk profile of sociodemographic factors can be appropriately routed to prehabilitation and behavioral counseling to prevent fatigue such as by intervening upon the modifiable predisposing (e.g., obesity) or precipitating factors.

Additionally, if routed to appropriate care early in the onset of fatigue, these patients may learn mitigating physical and cognitive-behavioral strategies [149,150,151]. Prehabilitation, a multidisciplinary clinical process on the continuum of care that occurs between diagnosis and treatment, involves targeted interventions to reduce the incidence and severity of future impairments [152]. For patients deemed at high risk for cancer-related fatigue, prehabilitation may beneficially include prescribed physical activity regimens, energy conservation techniques, and dietary consultations to prevent fatigue onset during and after treatment. Targeting pre-treatment windows for multimodal prehabilitation can help patients increase their physiologic reserve or functional capacity as early as possible, mitigating the effects of fatigue-inducing circumstances such as physical inactivity, inflammation, anorexia, and skeletal muscle loss. A recent, single-group study involving individualized, home-based aerobic and resistance training prior to breast cancer surgery demonstrated reduced fatigue over the intervention period [153]. More evidence, including randomized controlled trials of prehabilitation, is needed to confirm longer-term, post-treatment impact on fatigue. Multimodal prehabilitation interventions can also be delivered concurrently with cancer therapies, such as exercise during neoadjuvant chemotherapy or radiation therapy [154]. Prehabilitation may also profitably include cognitive behavioral therapy to reduce dysfunctional fatigue-related beliefs. If routed to appropriate care early in the onset of fatigue, patients can learn cognitive-behavioral strategies for optimizing sleep, emotion, and daily activity patterns in order to mitigate fatigue [149,150,151,155]. In fact, a recent study found that reduced fatigue in cancer patients was attributable to changes in cognition (specifically self-efficacy) stemming from cognitive behavioral therapy rather than to changes in physical activity [156]. Interventions targeting self-efficacy may therefore be particularly beneficial for patients with higher risk of fatigue.

After stratifying patients by risk based on predisposing (e.g., genetic) and perpetuating (e.g., diet, physical activity) factors, healthcare practitioners can leverage the 3P model by conducting ongoing screening for precipitating factors of cancer-related fatigue. These factors—such as metabolic dysregulation and inflammation—may arise at any point during cancer treatment, and can be watched closely using biomarker testing. Through monitoring for precipitating factors, providers can identify at-risk individuals who are not initially flagged for prehabilitation due to predisposing factors. These individuals would then receive prehabilitation appropriate for their specific needs, including specific exercise interventions optimally timed for each patient’s unique constellation of ability, challenges, and needs [157,158,159].

While being monitored for the emergence of new precipitating factors, cancer patients can concurrently receive education and training in health self-management techniques to mitigate symptoms of cancer-related fatigue. Health self-management is defined as “the individual’s ability to manage the symptoms, treatment, physical and psychosocial consequences and lifestyle changes inherent in living with a chronic condition” [160]. Crucially, self-management training can teach cancer survivors the tools they need to sustainably maintain physical activity, adhere to dietary recommendations, and exercise energy conservation and sleep hygiene techniques. These health behaviors reduce the risk of long-lasting fatigue after the conclusion of the treatment phase. Furthermore, research has demonstrated that symptoms may appear in a cascade pattern, and treating symptoms higher in the cascade may prevent downstream symptoms. For example, sleep disturbance contributes to fatigue, which in turn contributes to depressed mood. Interventions for cancer-related fatigue therefore may be specifically targeted to mitigate symptoms early in the cascade, such as sleep disturbance [161].

**Figure 2:**
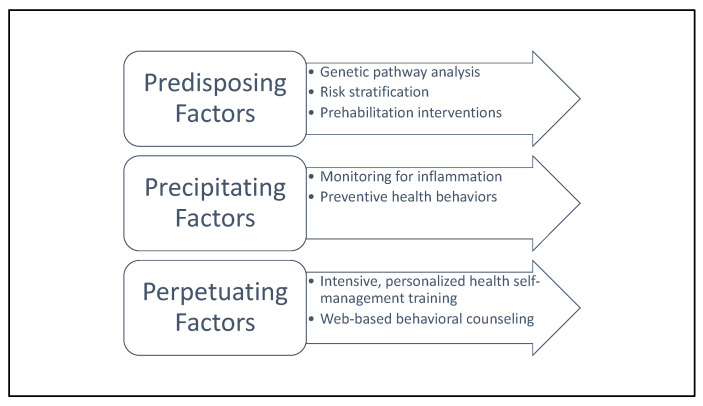
Recommended clinical actions to address each 3P factor.

Indeed, while clinicians may benefit from education about the 3P model in order to leverage its content for clinical practice, patients may also benefit from comprehensive education about the predisposing, precipitating, and perpetuating factors involved in cancer-related fatigue to promote sustainable, independent management of symptoms. Future health self-management training endeavors for cancer survivors may be enhanced by support from web-based health self-management training tools. This emerging area of practice provides patients with hyper-tailored health self-management plans, sometimes enhanced by machine-learning to be granularly responsive to the unique strengths and challenges of each person [162,163]. Please see Figure 2 for an overview of recommended clinical actions organized using the 3P model. Further research is needed to establish web-based tools powered by artificial intelligence specifically for the self-management of cancer-related fatigue.

## 5. Future Directions for Research

While clinical care can leverage the 3P model to prevent or mitigate symptoms of cancer-related fatigue, future research is also warranted to further investigate its pathophysiology and efficacy/effectiveness of adopting a 3P model in cancer care. One potentially fruitful target area for study may be the metabolome. The metabolome directly reflects the underlying biochemical activity and state of cells and tissues, including energy production. Because the metabolome is downstream of genomics, transcriptomics, and proteomics, it may be the closest molecular phenotype to the patient-reported phenotype of cancer-related fatigue. The metabolic state of an individual at the time of illness is produced by current environmental and host biological conditions, host susceptibility, and the aggregate history, time, and magnitude of exposures recorded as metabolic memory [164]. Moreover, preclinical research has recently identified inflammation-independent “metabolic reprogramming” as a mediator of cancer-induced fatigue in animal models [165,166]. Thus, the metabolome could provide a comprehensive snapshot of cellular processes at a single point in time that is also representative of cumulative exposures, ideal for discovery of new mechanisms of cancer-related fatigue.

The 3P framework proposed here to conceptualize cancer-related fatigue may also be applicable to fatigue related to other conditions such as chronic fatigue syndrome, post-COVID syndrome, rheumatoid arthritis, and multiple sclerosis. Through applying the 3P framework, researchers and clinicians studying fatigue across a wide variety of illnesses and chronic diseases may be able to more readily understand the pathophysiology of fatigue as it presents across various diagnoses, as well as systematically identify and mitigate predisposing, precipitating, and perpetuating factors relevant to unique populations experiencing fatigue.

## 6. Conclusions

For the growing number of cancer survivors worldwide, fatigue presents a major hurdle to a return to function and quality of life after cancer treatment. Treatment options for cancer-related fatigue are still emerging, and our current understanding of its etiology is limited. The presented 3P model may be leveraged—particularly using metabolomics, the microbiome, and inflammation in conjunction with behavioral science—to better understand the pathophysiology of cancer-related fatigue. While application of the 3P model alone will not be enough to solve the complex problem of cancer-related fatigue, it may represent a key step towards ameliorating this pervasive issue for patients. A clinical focus on organizing risk stratification and treatment around the 3P model would be warranted. Future research may benefit from expanding the 3P model to understand fatigue not only in oncology, but also in the wider context of a variety of chronic conditions.

## Figures and Tables

**Table 1:** 3P definitions, examples, and recommended clinical actions.

3P Component	Definition	Examples	Recommended Clinical Actions	
Predisposing Factors	Relatively stable patient characteristics that increase risk of developing cancer-related fatigue	Sex; age; genetics; circadian disruption; SNPS in circadian regulation; body composition; genetic variants altering metabolome and inflammasome	Genetic pathway analysis; risk stratification; tailored prehabilitation interventions	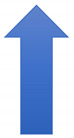 Patient education about the 3P model; standardized self-health-management techniques for mitigating cancer-related fatigue. 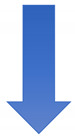
Precipitating Factors	States and traits that bring about or hasten the onset of cancer-related fatigue	Metabolic dysregulation; inflammation; biobehavioral: metabolic dysregulation, inflammation; treatment-related factors: systemic therapy, radiotherapy	Monitoring for inflammation; preventive health behaviors	
Perpetuating Factors	Characteristics and behaviors that worsen or prolong fatigue	Metabolic endotoxemia caused by changes in the microbiome; physical inactivity; sleep disturbance; biobehavioral: metabolic endotoxemia caused by changes in the microbiome, physical inactivity, circadian disruption, sleep disturbance. Treatment-related factors: maintenance therapy (e.g., aromatase inhibitors) Psychosocial: social isolation	Intensive, personalized health self-management training; web-based behavioral counseling	
